# Protein-Functionalized Microgel for Multiple Myeloma Cells’ 3D Culture

**DOI:** 10.3390/biomedicines10112797

**Published:** 2022-11-03

**Authors:** Juan Carlos Marín-Payá, Sandra Clara-Trujillo, Lourdes Cordón, Gloria Gallego Ferrer, Amparo Sempere, José Luis Gómez Ribelles

**Affiliations:** 1 Centre for Biomaterials and Tissue Engineering, CBIT, Universitat Politècnica de València, 46022 Valencia, Spain; 2 Biomedical Research Networking Center on Bioengineering, Biomaterials and Nanomedicine (CIBER-BBN), 28029 Valencia, Spain; 3 Centro de Investigación Biomédica en Red de Cáncer (CIBERONC), Instituto Carlos III, 20029 Madrid, Spain; 4 Hematology Research Group, Instituto de Investigación Sanitaria La Fe (IISLAFE), 46026 Valencia, Spain; 5 Haematology Department, Hospital Universitari i Politècnic La Fe, 46026 Valencia, Spain

**Keywords:** multiple myeloma, microgel, fibronectin, dexamethasone, bortezomib

## Abstract

Multiple myeloma is a hematologic neoplasm caused by an uncontrolled clonal proliferation of neoplastic plasma cells (nPCs) in the bone marrow. The development and survival of this disease is tightly related to the bone marrow environment. Proliferation and viability of nPCs depend on their interaction with the stromal cells and the extracellular matrix components, which also influences the appearance of drug resistance. Recapitulating these interactions in an in vitro culture requires 3D environments that incorporate the biomolecules of interest. In this work, we studied the proliferation and viability of three multiple myeloma cell lines in a microgel consisting of biostable microspheres with fibronectin (FN) on their surfaces. We also showed that the interaction of the RPMI8226 cell line with FN induced cell arrest in the G0/G1 cell cycle phase. RPMI8226 cells developed a significant resistance to dexamethasone, which was reduced when they were treated with dexamethasone and bortezomib in combination.

## 1. Introduction

Multiple myeloma (MM) accounts for approximately 10% of all hematological malignancies and 2% of all cancers [1,2]. It is characterized by the proliferation of neoplastic plasma cells (nPCs) in the bone marrow [2]. The development of a realistic 3D culture system is growing in relevance due to the important role played by the extracellular matrix (ECM) components and the stromal cells in their interactions with nPCs, promoting their survival and generating drug resistance [3,4,5,6]. Although new therapies have been developed, MM still remains an incurable disease due to the nPCs’ protection against chemotherapy or receptor-targeting drugs [7,8,9].

Tissue engineering has contributed to the development of 3D culture systems using inert materials, which acquire a bioactive capacity when functionalized with ECM biomolecules [10,11], promoting cell-material interaction and generating a similar response to that expected in vivo. The cellular and noncellular components of the tumor niche is the tumor microenvironment (TME), described by the International Cancer Microenvironment Society as an ecosystem composed of tumor cells, resident and infiltrating non-tumor cells, and molecules present in proximity to these cells. The ECM constitutes not only a structural scaffold but also plays a key role in the spread of cancers, as cell migration into and out of the TME greatly depends on the cell adhesion to ECM components. Tumor development is highly dependent on the physiological state of the tumor-specific microenvironment that will provide pro and antitumor signals [10,11,12].

In the case of multiple myeloma, it is demonstrated that the 3D support allows a closer emulation of the bone marrow environment than the conventional 2D cultures [12,13]. We have recently proposed the use of microgels for this purpose. The culture medium consists of an agglomerate of biomimetic microspheres and tumor cells suspended in a liquid culture medium [13,14]. Microspheres have frequently been used as drug delivery systems in the development of cancer models, and there are also references to their use as a three-dimensional support in the culture of solid tumors [15,16,17,18].

Recent and ongoing research has shown that properly recreating cell–cell and cell–ECM interactions is critical to developing reliable tumor models in vitro. We hypothesize that in the development of an in vitro disease model for multiple myeloma, the presence of ECM components presented by biomaterial in a cell-accessible manner is essential, while at the same time, the culture medium needs to allow the motility of cells that normally are grown in suspension. In this sense, our proposed biomimetic microgel would fulfill both requirements. In the future, it will be necessary to incorporate co-culture with adherent stromal cells, for which previous literature shows different strategies [6].

The correlation between tumor cell adhesion to fibronectin and the generation of drug resistance is well established [19]. In this work, we study the biological response to an environment formed by a microgel that exposes FN on the surface of microspheres and thus at distances from cells that are on the order of cellular dimensions. As the exhibition of adhesion motifs in FN is pivotal in cell–ECM interactions, mimicking the in vivo FN conformation when developing an artificial environment becomes of primary interest. Although cell activity produces FN fibrillogenesis in vivo modeling the ECM environment, certain biomaterials have been shown to induce FN spreading and networking on their surfaces previous to cell seeding. This feature is important in MM, since plasma cells are not expected to remodel the FN present on the surface of the supporting biomaterial used for cell culture. Physical absorption of FN onto poly(ethyl acrylate) has been demonstrated to preserve FN fibrillar networks and bioactivity by promoting their fibrillogenesis [20,21,22,23].

In cell–FN interaction, adhesion occurs through β1-integrins α4β1 (VLA-4) and α5β1 (VLA-5) (16). This adhesion induces a nuclear accumulation of p27kip1 proteins via down-regulation of Jab1 [7,24], preventing the serine 10 phosphorylation of p27Kip1, as well as the p27kip1 nuclear exportation. The resulting increase in the p27kip1 protein level inhibits cyclin A- and cyclin E-dependent CDK2 kinase activity [7,24,25], generating a G0/G1 cell cycle arrest and thus an intrinsic drug resistance to dexamethasone (DEX) in the RPMI8226 MM cell line [7], known as cell adhesion-mediated drug resistance (CAM-DR) [9,25].

Patients are currently treated in clinical practice with MM corticosteroids and proteasome inhibitors. The corticosteroid DEX induces apoptosis through the activation of intrinsic apoptotic pathways [26,27], downregulation of antiapoptotic genes, and upregulation of pro-apoptotic genes [26,28]. The recently introduced reversible inhibitor of the 26S proteasome complex bortezomib (BRZ) [29] induces the cleavage of survival proteins such as Mcl1 and triggers apoptosis [30,31]. In the RPMI8226 cell line, it has been found that BRZ overcomes the FN CAM-DR effect through the downregulation of VLA-4, and in combination with DEX, reduces the CAM-DR effect [29]. The drug dosage can be studied in MM patients by comparing the effect of a low-dose versus high-dose DEX [32,33]. Microgels, agglomerates of functionalized microspheres in a liquid culture medium, have also been proposed as a 3D environment for MM cell cultures [13,14]. 

This work aims to develop a 3D culture system based on a microgel made up of poly(ethyl acrylate) copolymers functionalized with ECM biomolecules, such as FN, and capable of mimicking both the bone marrow microenvironment by interacting with the tumor cells and the CAM-DR effect generated by the biomolecules. After validation, the system can be tested in vitro with primary cells from patients.

## 2. Materials and Methods

### 2.1. Polymerization and Microspheres Production

The microgel was made of a random ethyl acrylate (EA) and ethyl methacrylate (EMA) copolymer (Sigma-Aldrich, St. Louis, MO, USA), poly (EA-co-EMA) synthetized by a block polymerization protocol by mixing 50% EA and 50% EMA with 30% acetone *w*/*w* as a solvent. Initiation of the reaction was induced by 0.5% benzoin (Sigma-Aldrich) and a 24-h incubation of the solution in an ultraviolet (UV) irradiation chamber. After polymerization, the polymer was dissolved in acetone and reprecipitated four times with deionized water to remove the monomer residue and any remaining low molecular weight substances. The material was then dissolved in chloroform (3g/100 mL, Scharlab, S.L., Barcelona, Spain) and mixed with 5% *w*/*w* magnetic ferrite nanoparticles (EMG 1300M, Ferrotec, Santa Clara, CA, USA), facilitating the manipulation of the microspheres and their magnetic separation from cells or any aqueous medium.

The microspheres were produced by an oil-in-water emulsion technique, where the continuous phase was a 0.5% polyvinyl alcohol aqueous solution (PVA, Sigma-Aldrich), and the dispersed phase was a 3% *w*/*v* magnetic copolymer solution in chloroform. The emulsion was carried out in a volumetric flask by stirring 200 mL of the continuous phase at 2000 rpm for 15 min, adding 20 mL of the oil phase afterwards with the help of a funnel, and agitating for 10 min. Then, 150 mL of ultrapure water (UPW) was added to promote chloroform evaporation and stirred for 24 h. To complete chloroform evaporation overnight, the solution was later transferred to a beaker and stirred at 700 rpm. In order to remove the remaining PVA, the solution was transferred to 50 mL tubes by a series of four washings with UPW. After air drying the solution at room temperature, a field-emission scanning electron microscope (FESEM, ULTRA 55 model, ZEISS, Oberkochen, Germany) was used to observe the microspheres’ sizes and surfaces. The dispersion of the microspheres in culture conditions after manual stirring was confirmed in a stereo zoom microscope (MZ APO model, Leica Microsystems, Wetzlar, Germany). To determine microsphere sizes, 3 different emulsions were analyzed per condition (750, 1500, and 2000 rpm). At least 100 microspheres were measured for each emulsion, coming from 3 independent FESEM pictures. Image analysis was performed using the ImageJ software (National Institutes of Health, Bethesda, MD, USA).

### 2.2. Sterilization and Functionalization

A sterilization protocol was carried out, before using the microspheres for cell culture assays, by UV irradiation for 30 min and then incubating overnight in PBS with 3% penicillin/streptomycin. The day after, the microspheres were washed twice with PBS, UV-irradiated for 30 min, incubated overnight with PBS with 1% penicillin/streptomycin, and washed again with PBS. Once sterilized and before cell seeding, the microspheres were coated with 20 µg/mL of human plasma FN (Sigma-Aldrich) by physical adsorption for 1 h and washed twice with RPMI 1640 culture medium without FBS in order to functionalize them.

### 2.3. Characterization

The microspheres were characterized by a micro-BCA assay using a Pierce BCA Protein Assay Kit (Thermo Fisher Scientific, Waltham, MA USA). Acrylate microspheres without FN were used as a baseline. This assay was carried out in quadruplicate and read on a Victor3 Plate Reader (PerkinElmer, Waltham, MA, USA). Finally, the assessment was performed by calculating an average of the samples at 570 and 550 nm.

### 2.4. Cell Culture

The RPMI8226 MM cell line, kindly provided by Dr. Beatriz Martin (Josep Carreras Leukaemia Research Institute), was grown in an orbital shaker at 300 rpm in RPMI 1640 medium (Gibco, Thermo Fisher) and supplemented with 15% fetal bovine serum (FBS, Gibco), 1% l-glutamine (Sigma-Aldrich), and 1% penicillin/streptomycin (Gibco, 10.000 U/mL). The U226-B1 and MM.1S cell lines, purchased from the American Type Culture Collection (ATCC, Rockville, MD, USA), were grown as described above but supplemented with 10% FBS, 1% l-glutamine, and 1% penicillin/streptomycin the culture medium. Cell culture assays were performed under different conditions: cell suspension culture without microspheres (SUSP), cell suspension with uncoated microgel (M), and cell suspension with microgel coated with FN (MFN). Different microspheres/liquid-medium volume ratios were employed for the design of the 3D culture system in a final volume of 500 µL of microgels of 17.2% *v*/*v* microspheres (M17), 13.8% *v*/*v* (M13), 10.3% *v*/*v* (M10), and 6.8% *v*/*v* (M6). As it was a cell culture in suspension, a partial renewal of the liquid medium by adding 400 µL of fresh medium was performed every day by mixing for 15 minutes to facilitate the distribution of nutrients, precipitating cells and microgel by stopping the mixing after 1 h, and finally, carefully removing 400 µL of culture medium. Cell seeding was performed in triplicate on P24 plates. Both microgel and cell suspension were added consecutively in the proper ratios to obtain the desired microsphere volume fraction. In order to avoid the adhesion of the RPMI8226 cells to the other proteins present in the FBS (which can be adsorbed onto the microspheres’ surfaces) in the FN experiments, the cells were prior seeded in a culture medium without FBS for 1 h without mixing, and 1 h later, the appropriate amount of FBS was added to the wells. 

To perform flow cytometry assay and before cell staining, the cells and the magnetic acrylate microspheres were magnetically sorted out to prevent cytometer clogging.

### 2.5. Proliferation Assay

The influence on cell proliferation of the ratio of the volume occupied by the microspheres to that of the liquid culture medium was assessed using a Quant-iT Picogreen dsDNA Kit (Invitrogen, Thermo Fisher). For this, 5 × 10^4^ cells were grown at days 2, 5, and 7, including three replicas per condition. A lysis-buffered solution to digest the samples was prepared for 100 mL; amounts of 0.648 g of sodium phosphate monobasic (Panreac), 0.653 g of sodium phosphate dibasic (Panreac Quimica SLU, Barcelona, Spain), and 1 mL of EDTA (Gibco, 0.5 M) were dissolved in UPW at pH 6.5. Papain at 3.875 U/mL (Sigma-Aldrich) and 1.5 mg/mL of l-cysteine were added to the lysis buffer on the day of the experiment. For each sample, including the acrylate microspheres without cells as a baseline condition, 500 µL of lysis buffer was added and stirred at 60 for 18 h. Finally, DNA was stained with the PicoGreen reagent as indicated by the manufacturer and subsequently analyzed on an opaque Optiplate96F plate (PerkinElmer) using a Victor3 plate reader (PerkinElmer) at 485/535 nm.

### 2.6. Cell Cycle Assay and CAM-DR Effect on RPMI 8226 Cell Line

A flow cytometry cell cycle assay was performed using the DNA-PREP kit (Beckman Coulter, San Diego, CA, USA) to study the influence of adhesion of the RPMI8226 cells to FN on cell arrest. A total of 1.5 × 10^5^ cells were grown on days 2, 3, and 5, including three replicates per condition that were merged in a single tube and analyzed in the cytometer. Once available, the cells were washed twice with PBS at 300 g for 5 min in the centrifuge. Cells were processed with the DNA-PREP kit following the manufacturer’s instructions, which include a permeabilization step and staining with propidium iodide. The samples were acquired in a Navios flow cytometer and data were analyzed on the Kaluza Version 2.1 software (Beckman Coulter).

FN-induced CAM-DR was studied in the presence of BRZ and high-dose DEX. Three conditions were studied after growing 1.5 × 10^5^ cells to expose them to: (i) 5 nM of BRZ for 48 h [34,35], (ii) 10^3^ µM of DEX [14] at 8.75 mg/mL (fortecortin, Merck KGaA, Darmstadt, Germany) for 72 h, and iii) a DEX+BRZ combination, with only DEX for the first 24 h and both drugs for the last 48 h. A viability assay was carried out using annexin-V (Miltenyi Biotec, Bergisch Gladbach, Germany) and 7-amino-actinomycin D (7-AAD, Becton Dickinson, San Jose, CA, USA). Briefly, cells were washed twice with PBS, centrifuged at 300 g for 5 min, and resuspended in 300 µL of binding buffer solution (Miltenyi Biotec). Cells were stained with 10 µL of annexin-V conjugated with fluorescein isothiocyanate and 5 µL of CD138 monoclonal antibody conjugated with BD Horizon V500 (Becton Dickinson, San Jose, CA, USA), then incubated for 20 min at room temperature in the dark. Before staining with 7-AAD, the cells were washed with 1–2 mL of binding buffer solution and resuspended in 200 µL of binding buffer solution. Finally, the cells were stained with 10 µL of 7-AAD, incubated for 15 min at room temperature in the dark, and acquired in a FACSCanto-II flow cytometer (Becton Dickinson, San Jose, CA, USA). Data analysis was undertaken using Kaluza software.

### 2.7. Statistical Analysis

Statistically significant differences between groups were determined by applying one-way ANOVA analysis (Bonferroni test) on the IBM SPSS Statistics Version 20.0 software at a significance of ≤0.05.

## 3. Results

### 3.1. Acrylate Microsphere Production and Characterization

**Figure 1:**
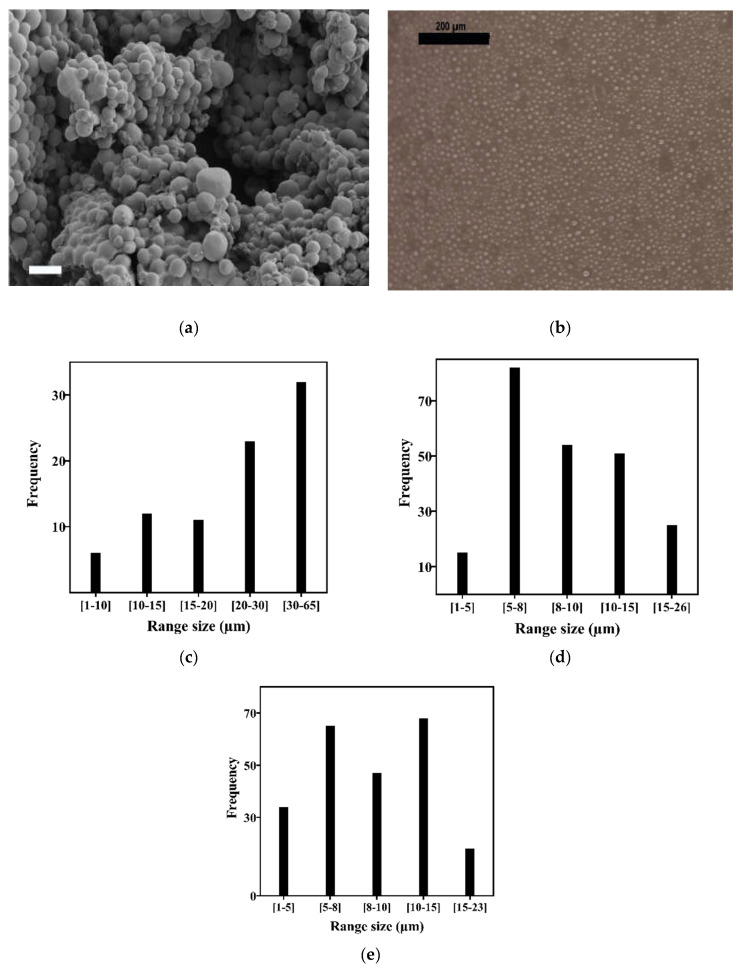
Physical microsphere characterization: (**a**) FESEM image of the microspheres (bar: 20 µm), (**b**) image of microgel dispersion in a P24 well, (**c**) size distribution at 750 rpm, (**d**) 1500 rpm, and (**e**) 2000 rpm.

Microgel microparticles were produced by an oil-in-water emulsion technique capable of controlling microdroplet size by varying the agitation speed. A representative FESEM image is shown in Figure 1a, while Figure 1b shows the dispersion of the microspheres in the liquid medium. The copolymer microsphere coagulation occurred due to the evaporation of the solvent throughout the continuous liquid phase. Three different tests were carried out at 750, 1,500, and 2,000 rpm agitation speeds. Figure 1 shows that as the stirring speed increased, the microsphere size decreased, obtaining an sizes of 27 ± 13 µm at 750 rpm (Figure 1c), 10 ± 4 µm at 1,500 rpm (Figure 1d), and 9 ± 4 µm at 2000 rpm (Figure 1e). The cell culture tests were performed using the conditions that provided a similar microsphere size to nPCs (9-20 µm) [36]. A 2000 rpm rotation speed was used, since the differences in the particle sizes with respect to 1500 rpm were small, but higher speeds favored chloroform evaporation, thus obtaining higher efficiency at the end of the process (data not shown).

Microspheres functionalized with FN by physical absorption were characterized by a micro-BCA assay to quantify the amount of FN present on them, for which coating was performed in triplicate. The mass fraction of FN was found to be 0.4 ± 0.07 µg per mg of microspheres.

The suspension of microspheres and cells was maintained under gentle orbital agitation. The characteristics of the environment of the cells cultured in this system were simulated by finite element analysis in a recent work [37].

### 3.2. Proliferation Assay

**Figure 2:**
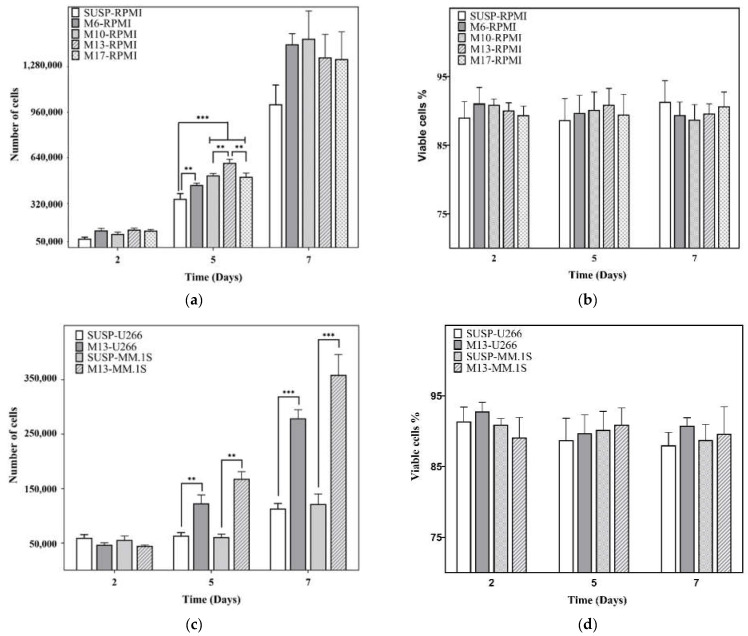
Evaluation of cell proliferation (**a**) and viability (**b**) on the RPMI8226 cell line at different volume ratios of uncoated microgels. Assessment of cell proliferation (**c**) and viability (**d**) on U266 and MM.1S cell lines at M13 volume ratio. SUSP: suspension culture without microspheres; M: suspension culture with different volume ratios of uncoated microspheres; M6: 6.89% *v*/*v* of microspheres; M10: 10.34% *v*/*v* of microspheres; M13: 13.8% *v*/*v* of microspheres; M17: 17.2% *v*/*v* of microspheres. Graphics depict mean ± standard deviation. Level of statistical significance: (**) *p*-value ≤ 0.01, (***) *p*-value ≤ 0.001.

The effect on cell proliferation of the volume occupied by the microspheres was tested at the beginning with the RPMI8226 cell line (Figure 2a). PicoGreen tests were performed on days 2, 5, and 7. On day 2, the differences between the microsphere volume ratios were not significant, nor were they statistically significant, even when the mean value was higher than in the SUSP-RPMI control. However, on day 5, all the conditions with microgel present were found to have significant differences *vs* SUSP-RPMI, especially the M13-RPMI, with a difference of 2.52 × 10^5^ ± 2.49 × 10^4^ cells, with respect to the SUSP-RPMI condition. On day 7, the number of cells reached a value close to 1.5 × 10^6^ cell/well (containing 500 µL of culture medium), which is the recommended limit for this cell line. The culture seemed to be saturated, and no significant differences were found between the samples. A viability assay was performed to confirm that there was no interference of the cell death with the PicoGreen analysis, obtaining around 88.9 ± 1.9% of cell viability under all conditions (Figure 2b). Based on these results, the M13 condition was used for the subsequent assays, and we determined whether this behavior was replicated in different multiple myeloma cell lines. Significant differences were found in both cell lines on days 5 and 7, with a difference on day 7 of 1.66 × 10^5^ ± 1.6 × 10^4^ cells of M13-U266 *vs* SUSP-U266 and 2.37 × 10^5^ ± 3.74 × 10^4^ cells of M13-MM.1S *vs* SUSP-MM.1S (Figure 2c). Due to the higher proliferation of RPMI8226 than U266-B1 and MM.1S, both cell lines did not reach the previous saturation level, while cell viability was around 90.2 ± 2.4% in U266 and 89.9 ± 2.3% in MM.1S (Figure 2d).

### 3.3. Cell Cycle Analysis on the RPMI 8226 Cell Line

**Figure 3:**
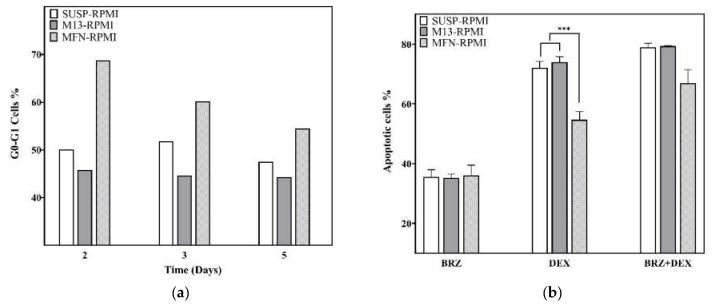
(**a**) Proportion of cells in the G0/G1 phase. (**b**) Results of drug resistance and the synergistic activity effect of both drugs on the RPMI8226 cell line viability. SUSP: suspension culture without microspheres. M13: suspension culture with 13.8% *v*/*v* of microspheres with uncoated microspheres. MFN: suspension culture with 13.8% *v*/*v* of microspheres coated with fibronectin. Graphic depicts mean ± standard deviation. Level of statistical significance: (***) *p*-value ≤ 0.001.

The adhesion of nPCs to FN was studied by a cell cycle assay on days 2, 3, and 5. As shown in Figure 3A, on day 2, the MFN-RPMI condition exhibited a higher arrest in the G0/G1 phase than the others, the differences being 18% and 23% with respect to the SUSP-RPMI and the M13-RPMI, respectively. On day 3, differences reduced to approximately 10% and 16% with respect to the SUSP-RPMI and the M13-RPMI, respectively. On day 5, there were differences of 7% and 10% regarding the SUSP-RPMI and M13-RPMI, respectively.

### 3.4. Drug Resistance on RPMI8226 Cell Line

To assess the CAM-DR effect of FN against high-dose DEX and BRZ, a flow cytometry viability assay was performed by preparing an untreated control for each condition that was used to normalize the viability results obtained by the effect of the drug (data not shown). After exposure to BRZ, only very similar results were observed in all the conditions, with a mortality of 35.5% in SUSP-RPMI, 35.2% in M13-RPMI, and 36% in MFN-RPMI (Figure 3b). In the only presence of DEX, the FN condition presented significant differences to SUSP-RPMI and M13-RPMI, showing a mortality of 54.6%, compared to 72% for SUSP-RPMI and 73.9% for M13-RPMI. In the presence of both drugs, there were very few differences between the SUSP-RPMI and M13-RPMI conditions, compared to those previously observed with only DEX, with a mortality of 78.8% SUSP-RPMI and 79.3% M13-RPMI. However, mortality increased by 12.2% in the FN condition with respect to that with only DEX, with a mortality of 66.8%.

## 4. Discussion

The development of 3D culture systems that can mimic and reproduce the tumor microenvironment and generate a similar cellular response to that produced in vivo is a common research goal at present [38,39]. However, to establish an in vitro model that provides conclusions about the effect of a certain factor on the generation of drug resistance is a real challenge. (In this work, we explored nPC adhesion to FN, but this can be easily extrapolated to interactions with other extracellular matrix components.) The environment in which the disease develops is highly complex, with several differentiated microenvironments and multiple cell–cell and cell–ECM component interactions with soluble factors [3,4]. When establishing a model aiming to isolate one of the factors that contributes to the development of the pathology, there is a risk of reaching erroneous conclusions due to the lack of other elements that affect the equation, in particular, the 3D configuration and cell mobility [38,40]. In this work, we therefore describe a model that aims to present the biomolecules to the tumor cells in a more realistic way than simply allowing the biomolecule to adsorb on the bottom of the culture well. The model described here has three fundamental characteristics: (1) it enables the mobility of tumor cells in a liquid medium, practically without restriction, as the microspheres that float along with them are equally mobile; (2) the proximity among the cells and the biomolecules under study, are at distances of the order of cell sizes with the chosen volume fraction of the microspheres; (3) the use of a synthetic support that induces a protein conformation that helps it display its adhesion domains, allowing interaction with cells or growth factors. Its versatility in this regard is enormous, and previous literature shows multiple examples of surface functionalization that can be applied to microspheres to study specific biomolecules [41].

We developed a 3D culture environment using polyacrylate microspheres with an average size close to nPCs. This microgel configuration favors cell–cell interaction and inter-microsphere cell mobility, which in turn, allows the cells to interact with the biomolecules on the microgel surface. One of the pitfalls of this type of system with similar cell-microgel size is the generation of background interference due to the presence of the microgel during sample processing or the possibility of clogging the flow cytometer during measurement. However, due to the microspheres’ magnetic properties, the cells can be separated from the material, making the platform easy to manipulate when performing cytometry cell assays.

The effect of microspheres on nPC proliferation depends on the volume occupied by the biomaterial and the liquid medium available for cell suspension. The results obtained suggest that in nPC culture conditions in the presence of a microgel, higher cell proliferation was observed in different multiple myeloma cell lines (Figure 2a,c), in good agreement with other studies [13]. This can be attributed to the configuration of the 3D culture platform, where the microspheres suspended in the liquid medium act as a scaffold. The volume excluded by the microspheres might have an increased cell–cell contact effect. On the other hand, microsphere mobility avoids the restrictions imposed when cells are cultured in scaffold pores or in a conventional hydrogel where the cells are confined, providing increased freedom for the expansion and proliferation of cells. Since the in vivo characteristics of tumoral cells include an elevated proliferation rate, we used the M13 condition, which provided higher proliferation than the other conditions (Figure 2a), to obtain an in vitro model as close as possible to the in vivo model. On day 7 of culture, we found that the different microgel conditions tended to produce similar numbers of cells (Figure 2a). This was due to the culture’s high cell proliferation and volumetric limitations, in which the saturated culture presented a cell density of around 2 × 10^6^ cells/mL, when the supplier’s recommendation was not to exceed 1 × 10^6^ cells/mL.

The presence of FN in the microspheres was confirmed by micro-BCA. RPMI8226 adhesion to FN, as well as its CAM-DR effect, had previously been studied in conventional 2D cultures, with FN adhering to the bottom of the well, resulting in nPC arrest in the G0/G1 phase of the cell cycle [7,24]. Prior to the CAM-DR assays, we therefore performed a cell cycle assay on days 2, 3, and 5 to confirm cell–FN interaction. On day 2 of culture, the MFN-RPMI condition had an increased cell percentage in the G0/G1 phase, compared to the other conditions (Figure 3a). This difference gradually decreased over time as the rest of the cells not attached to FN continued to proliferate and reduce the percentage of those arrested in the G0/G1 phase. After confirming that the culture in the microgel allowed the cells to interact with the functionalized biomolecule on the microspheres’ surfaces, the microgel was tested for interference with the drug assays by evaluating the CAM-DR effect of FN against high-dose DEX and BRZ, two of the drugs used against MM in clinical practice. The effect of FN was previously reported in the RPMI8226 MM cell line. As expected, FN did not trigger any type of resistance to BRZ, as all the conditions had the same percentages of mortality (Figure 3B). However, in the case of DEX, there were significant differences between the FN and the other condition because the arrest in phase G0/G1 was found to be associated with the generation of resistance to DEX [7]. Finally, we carried out an assay by combining the actions of both drugs for 72 h, incorporating only DEX for the first 24 h and DEX + BRZ in the last 48 h. The results obtained suggest that the action of BRZ is in some way masked by the high dose of DEX, with no differences in mortality between the SUSP-RPMI and M13-RPMI conditions and the only DEX condition. However, due to the downregulation of VLA-4 produced by BRZ [29], it was found that the MFN-RPMI condition, which showed an initial resistance to DEX, overcame this CAM-DR effect and presented higher mortality than the FN condition with only DEX, confirming that the resistance was generated by the CAM-DR effect of FN.

This work was limited to verifying the viability and proliferation of the multiple myeloma cell line RPMI8226 in the microgel and the effect of fibronectin on resistance to dexamethasone and bortezomib, in order to show the capability of our 3D environment in the study of multiple myeloma. Future developments will be aimed at developing microgels exhibiting other biomolecules of the bone marrow ECM on the surfaces of the microspheres. The resistance of primary tumor plasma cells to the drugs currently used in tumor treatment will be studied.

## 5. Conclusions

We have shown that it is possible to consolidate the development of a 3D culture platform based on microgels. The system, easy to manipulate, could be proposed as a very useful strategy in clinical practice to test single agents or drug combinations that can produce a synergistic effect by increasing the anti-tumoral potential [30] for the treatment of hematological neoplasms. This work offers a wide range of future research perspectives, such as the development of 3D cultures with other ECM components or co-cultures with stromal cells. The validation of this platform for the culture of MM primary cells from patients could be applied to test and develop novel drugs for a better understanding of their effects and the interaction between tumoral cells and the bone marrow environment.

## Data Availability

Publicly available datasets were analyzed in this study. This data can be found at the Riunet repository of the Universitat Politècnica de València, here: [ http://hdl.handle.net/10251/189950].
